# Elucidating the role of brassinosteroid signaling genes and their promoters in *Arabidopsis* revealed regulatory mechanisms in plant development and responses to different abiotic stresses

**DOI:** 10.1186/s12870-025-06960-6

**Published:** 2025-07-28

**Authors:** Sunny Ahmar, Muhammad Sohaib Shafique, Marcin Rapacz, Ewa Pociecha

**Affiliations:** 1 Department of Plant Breeding, Physiology and Seed Science, Faculty of Agriculture and Economics, University of Agriculture in Krakow, Podłużna 3, Krakow, 30-239 Poland; 2 State Key Laboratory of Crop Gene Resources and Breeding, National Key Facility for Crop Gene Resources and Genetic Improvement, Institute of Crop Sciences, Chinese Academy of Agricultural Sciences, Beijing, 100081 China

**Keywords:** Brassinosteroids, BR signaling pathway, Plant architecture, BR signaling genes, Regulatory mechanisms

## Abstract

Brassinosteroids (BRs) are essential plant steroid phytohormones that regulate a wide range of developmental processes and mediate plant responses to various biotic and abiotic stresses. BR signaling regulators have significant potential to optimize plant architecture and enhance resilience to environmental stress. Although the BR signaling pathway is well-characterized in the dicot model species *Arabidopsis* (*Arabidopsis thaliana*), our knowledge of this pathway at the transcriptional level remains limited and requires further investigation. Moreover, the functional roles of several other conserved genes involved in the BR signaling pathway in *Arabidopsis* are not yet fully understood. Therefore, the present study was designed to conduct detailed and comprehensive analysis of BR signaling genes, promoter regions, and their encoded proteins in *Arabidopsis*. A bibliometric approach was used to compile a list of potential BR-signalling genes. A total of 41 BR signaling genes and their promoter sequences were selected for comprehensive *silico* analyses, including gene structure visualization, characterization, phylogenetic evolution, identification of cis-regulatory elements, and prediction of different transcription factor binding sites (TFbs) within 1.5 kb upstream of the promoter sequences in *Arabidopsis.* The expression patterns of these 41 BR signaling genes were examined in various plant organs and under different abiotic and hormonal stress conditions. Quantitative PCR (qPCR) was performed to validate the expression profiles of several BR signaling genes under osmotic stress (polyethylene glycol (PEG)-induced) and salt stress. In addition, protein-protein interactions (PPI) encoded by BR signaling genes were predicted in *Arabidopsis*. These analyses identified different types and frequencies of cis-elements and TFbs associated with plant growth and stress responses. Overall, this study provides valuable insights into the regulatory mechanisms underlying the coordinated expression of BR signaling genes in *Arabidopsis*, with potential implications for both monocots and dicots.

**Supplementary Information:**

The online version contains supplementary material available at 10.1186/s12870-025-06960-6.

## Introduction

Brassinosteroids (BR) are naturally occurring phytohormones that regulate various agronomic traits and enhance plant resilience to biotic and abiotic stresses. Furthermore, BRs are recognized as promising tools for maintaining sustainable agricultural productivity, and BR-related genes have the potential to drive the modern green revolution [1, 2]. The loss of function of BR signaling or biosynthesis genes often leads to severe disturbances in plant architecture, including dwarfism or semi-dwarfism, dark green leaves, photomorphogenesis under dark conditions, and delayed flowering [3]. Therefore, BR homeostasis is essential for optimal plant growth and development [4]. Over the last three decades, extensive genetic and biochemical studies have comprehensively described the BR signaling pathway, particularly in the model dicot plant *Arabidopsis thaliana*, making it one of the best characterized molecular signaling pathways in plants [5, 6]. The BR signaling pathway begins with the membrane-localized receptor *BRASSINOSTEROID INSENSITIVE 1 (BRI1)*, which forms a complex with its co-receptor *BRI1-ASSOCIATED RECEPTOR KINASE 1 (BAK1)*, leading to the activation of the transcription factors *BRI1-EMSSUPPRESSOR (BES1)* and *BRASSINAZOLE-RESISTANT 1 (BZR1).* These transcription factors (TFs) constitute a complex transcriptional network that governs the expression patterns of thousands of genes by binding to cis-regulatory elements in the promoter regions of target genes, thereby modulating plant architecture and stress responses [7–14].

Several members of different protein families redundantly regulate various steps in the BR signaling pathway. These include BR receptors (*BRI1*,* BRL1*, and BRL3) and their co-receptors (*SERK1*,* SERK3/BAK1*,* SERK4*,* and SERK5).* In addition, *GSK3-like kinases (*BIN2, ASKι, and ASKζ) are key negative regulators that modulate signal transduction [15, 16]. Furthermore, two major TFs, *BES1* and *BZR1*, and their homologs, *BEH1 1–4*, form a small TF family that coordinates BR-mediated gene expression during downstream events [14, 17, 18]. Functional redundancy among these signaling components enhances the robustness of the BR pathway, which continues to function even when some BR components are impaired. The complexity of the BR signaling pathway often makes it difficult to understand the specific roles of individual components and the relationships among different protein families [14]. However, most BR signaling genes in *Arabidopsis* have been functionally characterized using forward and reverse genetic approaches [19, 20]. To date, most studies have focused on the interaction between *BES1* and *BZR1* TFs and their downstream target genes, whereas knowledge about other conserved genes in the BR signaling pathway is limited, even in *Arabidopsis* and the monocot model plant (*Oryza sativa*) [21]. However, several genes involved in the BR signaling pathway function independently and regulate diverse plant developmental processes beyond classical BR responses [22].

The BR signaling pathway exhibits notable differences between monocots and dicots. For instance, several BR signaling components, such as *Protein Phosphatase 2 A (PP2A)* and *BRI1-Supressor1 (BSU) phosphatases* identified in *Arabidopsis*, do not have orthologs in rice. Similarly, some BR signaling components are only present in rice and require further investigation in *Arabidopsis.* These observations suggest that specific components may have specific functional roles in monocots and dicots or contribute to redundancy within the signaling network [23–26]. To date, 100 BR signaling components have been identified in *Arabidopsis*, compared to approximately 39 in rice, although efforts to discover and characterize additional BR signaling components in both species are ongoing. Despite significant progress, there is limited information regarding the transcriptional regulation of BR signaling genes in *Arabidopsis* and rice [27]. To address this gap and consider the complexity of the BR signaling pathway, a recent study conducted a detailed and comparative analysis of BR signaling genes and their promoter sequences in rice [28]. These analyses provide important insights into the regulatory mechanisms through which BR signaling genes control gene expression during plant development and mediate responses to different environmental stresses [28].

Previous studies on *Arabidopsis* have indicated that in silico analysis of different gene families and their promoters provides valuable insights into how plants coordinate developmental processes and stress responses at the transcriptional level. For example, promoter analyses of the *Bcl-2 athanogene (BAG)* gene family [29], *pathogenesis (PR) genes* [30], and dehydration response element binding factor (DREBs) genes [31] have revealed the presence of various types of cis-elements and TFbs in their promoter regions, suggesting their potential roles in different plant organs and responses to environmental stresses. However, no detailed comparative analyses of BR signaling genes have been conducted in *Arabidopsis*. The BR signaling genes in *Arabidopsis* belong to the same genes families and often exhibit similar Functions, making it difficult to understand their specific roles in plant development.

**Fig. 1:**
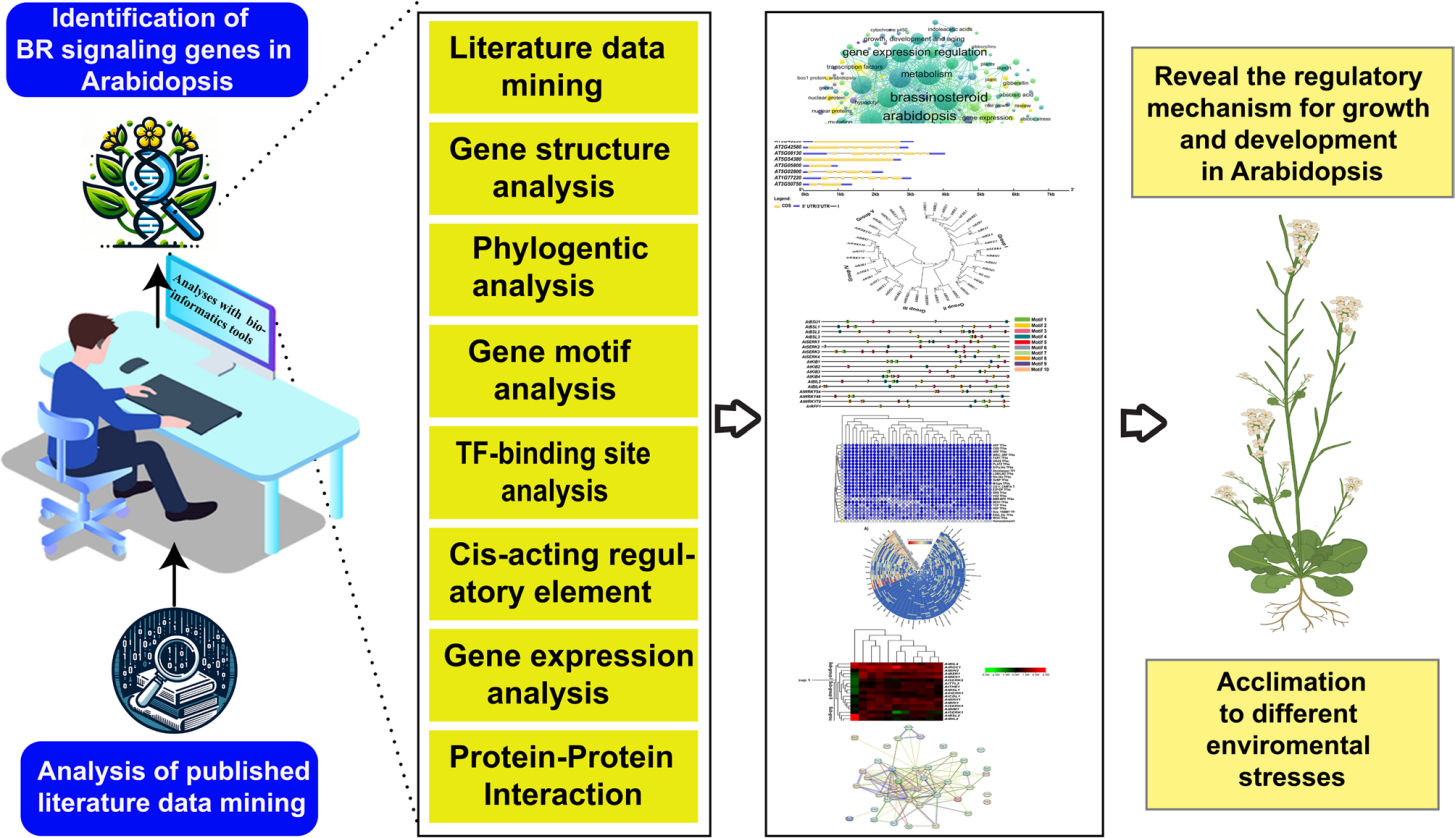
A systematic overview of the current study

Furthermore, findings from previous studies suggest that in-depth gene analyses of distinct promoter sequences associated with various biological processes in plants could provide valuable insights into the mechanisms that regulate gene expression and its coordinated action during plant development and in response to environmental stimuli [29–31]. Therefore, this study aimed to perform a detailed and comparative analysis of BR signaling genes in *Arabidopsis* to reveal their regulatory mechanisms in different plant organs and their responses to environmental stresses (Fig. 1). These analyses may provide insights into the distribution and abundance of cis-regulatory elements and TFbs in promoter regions. The identification of potential cis-elements and TFbs will contribute to further investigation to reveal their putative roles in coordinating the expression of BR signaling genes during plant growth, development, and stress responses. These findings could lead to the development of novel strategies for enhancing crop resilience and productivity by manipulating BR signaling pathway.

## Material and method

### Plant materials

Seeds of *Arabidopsis* ecotype Columbia (Col-0) were obtained from the Center of Crop Germplasm Resources, Institute of Crop Sciences (Beijing, China). All plant materials were maintained, and experiments were conducted at the Institute of Crop Sciences, Chinese Academy of Agricultural Sciences (CAAS) in Beijing, China.

### Data collection and selection of BR signaling gene in *Arabidopsis* by literature mining

We performed a bibliometric analysis using the Scopus database to select BR-signaling genes in *Arabidopsis*. Scopus is a comprehensive scientific database that provides extensive coverage of peer-reviewed literature, including references and citations [32]. Our search was performed within the article title, keywords, and abstract sections using the following query: Brassinosteroid AND signaling AND genes AND Arabidopsis. Subsequently, we downloaded the output data and performed various analyses using the bibliometric package R Studio (version 4.1.2). To assess research trends and thematic focus, we examined the total number of publications per year from (to 1997-April 2024). In addition, a map of co-occurrence keywords was generated to verify prominent terms and confirm their relevance to the search query.

Furthermore, a topic trend analysis was conducted to identify the top keywords associated with BR signaling. Based on this analysis, we selected the top-ranking keywords that identified candidate genes involved in BR signaling. This approach identified 117 genes that play direct or indirect roles in the BR signaling pathway. A total of 117 gene IDs with documented direct or indirect roles in BR signaling were identified. Principal component analysis (PCA) was performed using keywords based on correlations to refine the gene list. Consequently, 41 genes were shortlisted for detailed in silico analyses.

### Retrieval and visualization of the sequences of selected BR-signaling genes in *Arabidopsis*

This study analyzed the 1.5kb upstream promoter regions, full-length genomic, CDS, and corresponding protein sequences of 41 BR signaling genes in *Arabidopsis* involved in the BR signaling pathway. These sequences were retrieved from the Arabidopsis Information Resource (TAIR) (https://www.arabidopsis.org/). Furthermore, we cross-checked the genes, transcripts, and protein lengths of each BR signaling gene and the sequence start and end points using Phytozome v13 157 (http://phytozome-next.jgi.doe.gov/) and National Center for Biotechnology Information (NCBI) databases. The exon-intron structures of the BR signaling genes were visualized using the Gene Structure Display Server 2 (GSDS2) (http://gsds2.cbi.pku.edu.cn).

### The retrieval and phylogenetic analysis of 1.5kb upstream promoter sequences and protein sequences

The 1.5 kb promoter and protein sequences of BR signaling were aligned using Geneious 4.8.5 software and MAFFT with the ‘L-INS-i algorithm. To assess the presence of phylogenetic signals, we used IQ-TREE 2 with 3000 quartets under the TEST model without tree reconstruction and applied likelihood mapping analysis to determine the phylogenetic signals in the promoter and protein sequences. We checked for phylogenetic signals using the following parameters: if the unresolved ratio exceeded 30%, it was considered to have a weak phylogenetic signal. Sequence divergence heterogeneity was evaluated using the AliGROOVE method (Kück et al. 2014). Furthermore, phylogenetic analyses and the neighbor-joining method were performed using MEGA 7 software with a p-distance model. The robustness of each clade was assessed using bootstrap analysis with 1000 replicates. The plots were generated using the R packages “ggtree,” “treeio, and “ggplot2 within R-studio.

### Identification of conserved motif analysis and common cis element within 1.5kb promoter region

The 1.5 kb upstream promoter sequences of 41 BR signaling genes in *Arabidopsis* were analyzed using the MEME tool (http://meme-suite.org/index.html) to identify conserved motifs. Up to ten conserved motifs were detected in each sequence. The identified motifs were visualized using TBtools v0.6655. Furthermore, we identified putative cis-elements within 1.5 kb upstream of the promoter sequences using the PlantCARE database (http://bioinformatics.psb.ugent.be/webtools/plantcare/html/). A heatmap representing the distribution of common cis-elements across BR signaling gene promoters was generated using TB tools. A Circos diagram illustrating the spatial distribution of these elements was created using the Circos table viewer online tool (https://mk.bcgsc.ca/tableviewer/visualize/).

### Identification and visualization of common TFbs sites within the 1.5 kb promoter region

The PlantPAN 4.0 online tool (http://plantpan.itps.ncku.edu.tw/promoter.php) was used to identify transcription factor binding sites (TFbs) within the 1.5 kb upstream promoter sequences of the 41 BR signaling genes. A multiple promoter analysis program was used to determine the frequency and distribution of TFbs across the promoter region (http://plantpan2.itps.ncku.edu.tw/gene_group.php?#multipromoters). The resulting data were visualized as heatmaps using TBtools v0.6655. A correlation matrix heatmap was generated using Python (version 3.10.10).

### Expression pattern of selected BR signaling genes and qTR-PCR analysis

The expression patterns of 41 BR signaling genes in *Arabidopsis* were analyzed across various plant organs and in response to different hormones and abiotic stresses. Transcriptomic data was retrieved using an online public database named the Plant Public RNA-seq Database (http://ipf.sustech.edu.cn/pub/plantrna/). Tissue-specific expression was examined across various plant organs, including the endosperm, flowers, hypocotyls, leaves, roots, seeds, cotyledons, inflorescences, pollen, shoots, and siliques. In addition, gene expression responses to exogenous hormone treatments, such as abscisic acid (ABA), ethylene, and gibberellic acid (GA), were analyzed and compared with those under control conditions. Furthermore, expression data in response to abiotic stresses, such as drought, salt, heat, and cold, were analyzed and compared to control conditions. The expression data were used to perform a hierarchical clustering analysis of the complete method using log2 values. Heatmaps were generated using TBtools v0.6655, with red, black, and green color gradients to visualize the differential expression levels.

From the set of 41 BR-signaling genes, 12 BR-signaling genes (*AtBRI1*,* AtSERK3*,* AtBZR1*,* AtBIN2*,* AtBEH*,* AtBES1*,* AtBSL1*,* AtKIB1*,* AtWRKY54*,* AtBEE1*,* AtBIL2*,* and AtBKI1*) were selected for their expression patterns. These genes were prioritized based on their central regulatory roles in the BR signaling cascade, including receptor perception, signal transduction, and transcriptional regulation. We validated the expression of these genes in *Arabidopsis* (Col-0 ecotype) under salt and osmotic stress (polyethylene glycol (PEG)-induced) conditions using qRT-PCR. *Arabidopsis* seeds (Col-0) were stratified at 4 °C for 2 days and grown in a controlled growth chamber under long-day conditions (16 h light/8 h dark) at 22 °C, 60% relative humidity, and a light intensity of 120 µmol m⁻² s⁻¹. Salt and osmotic (PEG-induced) stress were induced in Pindstrup soil-grown plants (four-week-old seedlings) using 150 mM NaCl and 15% PEG 6000. A 24-hour post-treatment, leaf samples were collected for RNA extraction. Total RNA was extracted using RNAiso Plus (TaKara Bio Inc., Japan) following the manufacturer’s protocol with minor modifications, including an extended lysis time (10 min) and an additional chloroform extraction step. RNA quality and integrity were confirmed using agarose gel electrophoresis and Nanodrop spectrophotometry. First-strand cDNA synthesis was performed using the FastKing RT Kit (TIANGEN Biotech, China). Quantitative real-time PCR (qRT-PCR) was performed using primers listed in Supplementary File S1.

### Protein-protein interaction encoded by 41 BR signaling proteins

Protein-protein interactions (PPI) were predicted for 41 BR signaling proteins using the online web tool STRING online platform (https://string-db.org/) with a confidence level of 0.7. Functional enrichment analysis of the interactome was performed at a significance level of *p* < 0.01 [28].

## Results

### Selection of BR signaling genes through bibliometric analysis

**Fig. 2:**
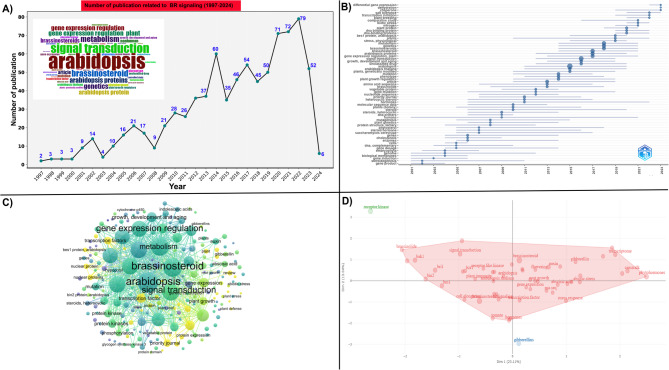
Bibliographic approach for the selection of BR signaling genes in *Arabidopsis*. **A **Number of articles published between January 1997 and April 2024 with the most representative keywords “brassinosteroid” and “signal transduction” in *Arabidopsis*. **B** The most trending topics showed interconnections owing to the presence of the highest frequencies within published articles. **C** Map of co-occurrence based on keywords from published articles. **D** Principal component analysis (PCA) and its positive and negative correlations with BR and other keywords

The bibliometric analysis identified 833 articles published between January 1997 and April 2024 that focused on BR signaling. The most common keywords represented in these articles were *Arabidopsis*, brassinosteroid, and signal transduction, as shown in the materials and methods section and visualized in Fig. 2A. From 2011 to 2023, the number of articles on BR signaling in *Arabidopsis* increased, indicating growing scientific interest in investigating the role of BR signaling in plants (Fig. 2 A). Furthermore, topic trends were assessed based on the frequency of terms in the titles, abstracts, and keywords of the articles. The keywords *Arabidopsis*, signal transduction, brassinosteroid, and stress physiology had the highest frequencies among these articles, suggesting a strong interconnection between these themes (Fig. 2B). Similar patterns were observed on the map of co-occurrence keywords (Fig. 2C), which facilitated the retrieval of lists of genes related to BR signaling in *Arabidopsis*. A total of 117 genes related to BR signaling were identified. These potential genes were cross-checked with TAIR and NCBI to verify their functional relevance in BR signaling in *Arabidopsis*.

However, to reduce the number of genes for in-silico analysis, PCA was conducted using bibliometric data based on 117 BR-related genes. PCA revealed both positive and negative correlations between BR signaling and several thematic keywords, such as stress responses and plant hormones, confirming the multiple roles of BR in the plant life cycle (Fig. 2D). Based on PCA associated with other bibliometric data, 41 core BR signaling genes were selected for analysis. These genes are functionally relevant, either directly or indirectly, to the BR signaling pathway during plant developmental stages and in response to different environmental stresses (Table S1).

### Analysis and characterization of selected BR signaling genes in *Arabidopsis*

The number and position of exons and introns are critical factors in determining the gene structure. We visualized the number and size of exons-introns across the selected BR signaling gene set in *Arabidopsis* to assess the evolutionary influence on the gene structure. Based on the results, the exon-to-intron ratios ranged from 22:21 to 1:0, indicating substantial structural diversity (Figure. S1). The total length of the BR signaling genes ranged from the longest, 7584 bp (*AT1G08420*,* AtBSL2*), to the shortest, 647 bp (*AT1G74500*,* AtBS1*) (Table 1). Further structural and functional details of these BR signaling genes and their encoded enzymes are shown in Figure S1 and Table 1, respectively.

**Table 1:** Characteristics of BR signaling genes and their encoded proteins in *Arabidopsis*

Gene name	Gene ID	Chr. #	Start (bp)	End (bp) Strand	Genomic length (bp)	Transcript length (bp)	CDS Length(bp)	Exons: Introns	Protein length (aa)
*AtBSU1*	*AT1G03445*	Chr1	854,409	859,701	5292	2727	2382	22:21	794
*AtBSL1*	*AT4G03080*	Chr4	1,359,654	1,365,475	5821	3235	2646	21:20	882
*AtBSL2*	*AT1G08420*	Chr1	2,649,536	2,657,120	7584	3740	3057	21:20	1019
*AtBSL3*	*AT2G27210*	Chr2	11,629,776	11,636,795	7019	3816	3021	21:20	1007
*AtSERK1*	*AT1G71830*	Chr1	27,018,157	27,022,117	3960	2570	1878	11:10	626
*AtSERK2*	*AT1G34210*	Chr1	12,458,669	12,462,905	4236	2448	1887	11:10	629
*AtSERK3*	*AT4G33430*	Chr4	16,086,310	16,090,777	4467	2821	1989	12:11	663
*AtSERK4*	*AT2G13790*	Chr2	5,741,819	5,746,793	4974	2234	1863	11:10	621
*AtKIB1*	*AT4G12810*	Chr4	7,522,830	7,523,979	1149	1149	1149	1:0	383
*AtKIB2*	*AT4G12820*	Chr4	7,527,047	7,529,356	2309	1552	1329	2:1	443
*AtKIB3*	*AT1G67160*	Chr1	25,124,818	25,127,098	2280	1353	1353	3:2	451
*AtKIB4*	*AT3G03730*	Chr3	935,138	936,320	1182	1182	1182	1:0	394
*AtBIL2*	*AT2G42080*	Chr2	17,553,239	17,555,504	2265	990	792	6:5	264
*AtBIL4*	*AT3G63310*	Chr3	23,387,855	23,388,985	1130	905	720	4:3	240
*AtWRKY54*	*AT2G40750*	Chr2	17,000,453	17,002,468	2015	1337	1041	3:2	347
*AtWRKY46*	*AT2G46400*	Chr2	19,043,413	19,044,826	1413	1222	888	3:2	296
*AtWRKY70*	*AT3G56400*	Chr3	20,908,927	20,910,481	1554	1111	885	3:2	295
*AtBEE1*	*AT1G18400*	Chr1	6,331,397	6,333,743	2346	1016	783	6:5	261
*AtBEE2*	*AT4G36540*	Chr4	17,243,582	17,245,296	1714	1362	915	5:4	305
*AtBEE3*	*AT1G73830*	Chr1	27,759,972	27,761,588	1616	939	786	6:5	262
*AtBRI1*	*AT4G39400*	Chr4	18,324,660	18,328,826	4166	4166	3591	1:0	1197
*AtBKI1*	*AT5G42750*	Chr5	17,142,819	17,144,160	1341	1341	1014	1:0	338
*AtBZR1*	*AT1G75080*	Chr1	28,185,503	28,188,075	2572	1563	1011	2:1	337
*AtBIN2*	*AT4G18710*	Chr4	10,296,272	10,301,228	4956	1793	1143	10:9	381
*AtBES1*	*AT1G19350*	Chr1	6,688,521	6,690,414	1893	1465	1074	3:2	358
*AtBRL2*	*AT2G01950*	Chr2	440,635	444,276	3641	3641	3432	1:0	1144
*AtBRH1*	*AT3G61460*	Chr3	22,741,510	22,742,284	774	774	513	1:0	171
*AtBIA1*	*AT4G15400*	Chr4	8,811,927	8,813,478	1551	1551	1308	1:0	436
*AtBRS1*	*AT4G30610*	Chr4	14,944,128	14,948,605	4477	1702	1398	9:8	466
*AtROC1*	*AT4G38740*	Chr4	18,083,394	18,084,251	857	857	519	1:0	173
*AtBZS1*	*AT4G39070*	Chr4	18,204,854	18,206,509	1655	1023	729	2:1	243
*AtBS1*	*AT1G74500*	Chr1	27,998,174	27,998,821	647	558	282	2:1	94
*AtBPG2*	*AT3G57180*	Chr3	21,163,544	21,166,138	2594	2232	1980	4:3	661
*AtHERK1*	*AT3G46290*	Chr3	17,012,727	17,015,885	3158	3032	2493	1:0	831
*AtTTL3*	*AT2G42580*	Chr2	17,728,714	17,731,718	3004	2473	2076	7:6	692
*AtBIM1*	*AT5G08130*	Chr5	2,606,199	2,610,250	4051	2553	1599	11:10	533
*AtTHE1*	*AT5G54380*	Chr5	22,077,091	22,079,880	2789	2789	2568	1:0	856
*AtAIF1*	*AT3G05800*	Chr3	1,727,305	1,728,296	991	991	636	1:0	212
*AtCDL1*	*AT5G02800*	Chr5	635,227	637,503	2276	1583	1137	5:4	379
*AtLAZ1*	*AT1G77220*	Chr1	29,012,716	29,015,803	3087	2025	1455	8:7	485
*AtBEH1*	*AT3G50750*	Chr3	18,861,838	18,863,234	1396	1311	831	2:1	277

### Phylogenetic analysis selected BR signaling genes

**Fig. 3:**
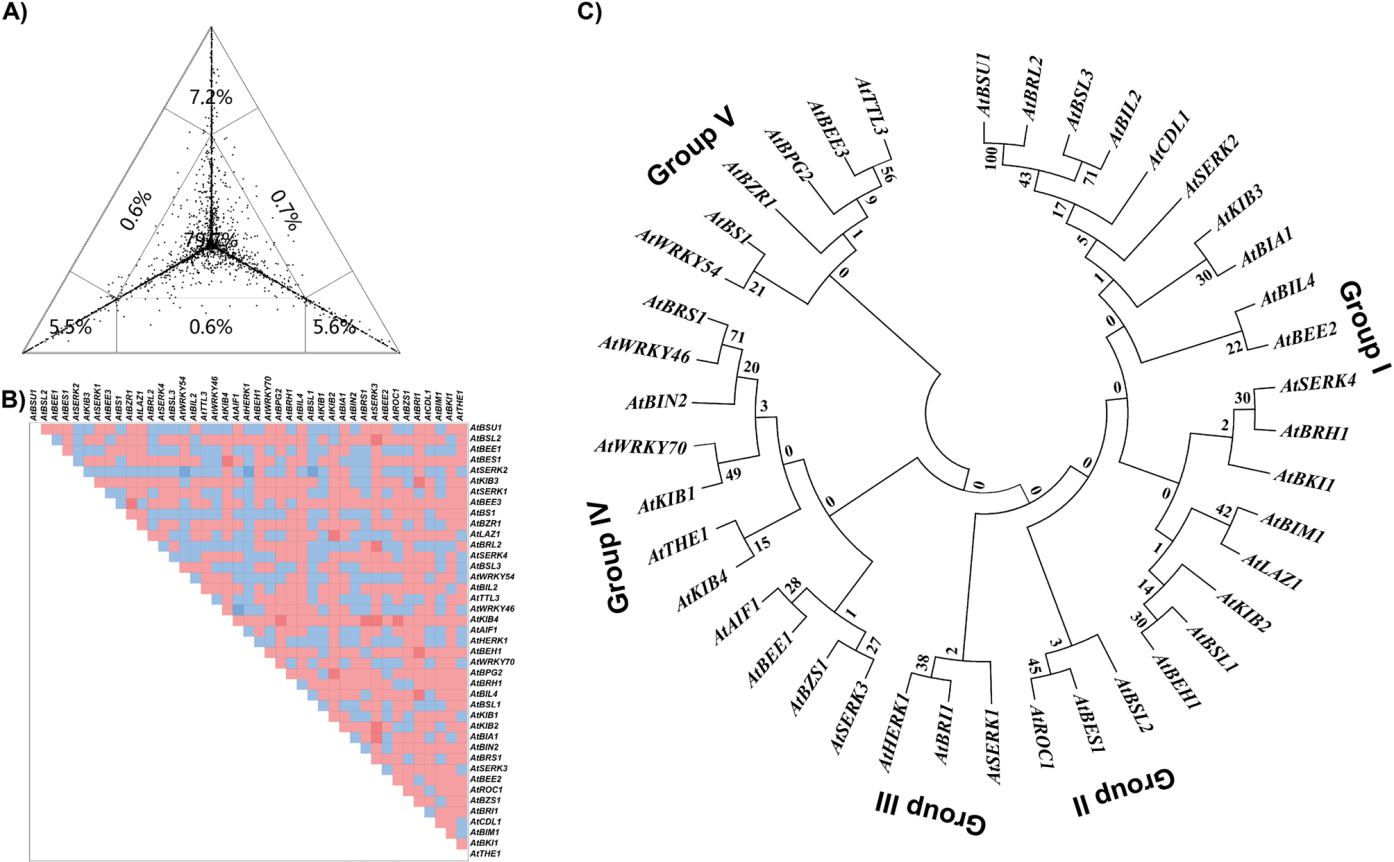
Phylogenetic analysis of 1.5 kb upstream promoter sequences of selected BR signaling genes. **A** detailed and comparative analysis of BR signaling genes revealed key insights into the structural, reg) Phylogenetic signal test. **B** Heterozygosity variation within the 1.5 kb upstream promoter sequences **C** Phylogenetic tree of the 1.5 kb upstream promoter sequences of selected BR signaling genes

**Fig. 4:**
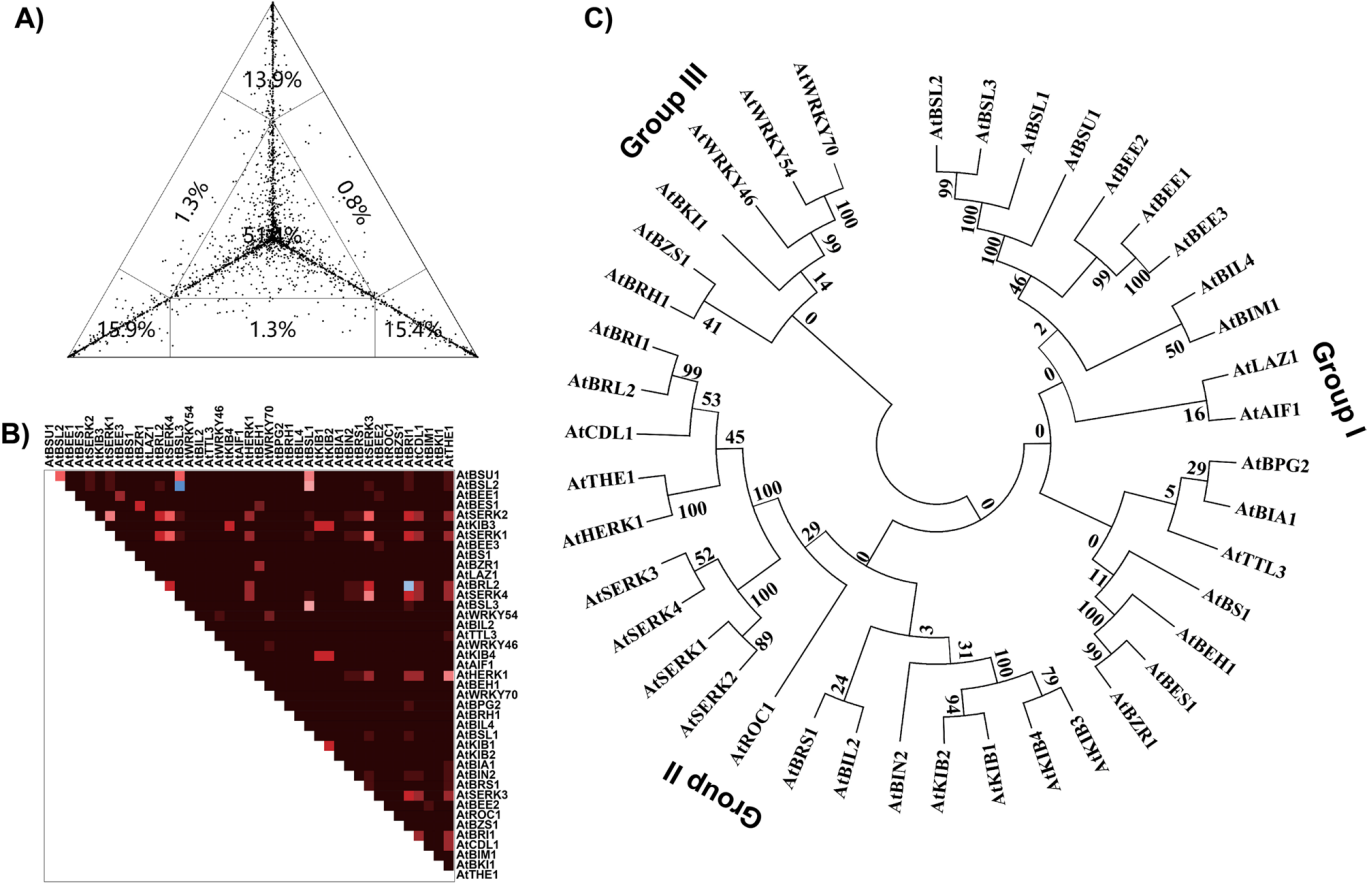
Phylogenetic analysis of amino acid sequences of selected BR signaling genes. **A** Phylogenetic signal test. **B** Heterozygosity variation within amino acid sequences (**C**) Phylogenetic tree of amino acid sequences

In this study, we conducted a comparative phylogenetic analysis of 1.5 kb upstream promoter regions and amino acid sequences of selected BR signaling genes in *Arabidopsis*. First, a phylogenetic signal test was performed to check the suitability of these sequences for phylogenetic analysis (Figs. 3A and 4A). The analysis revealed weak phylogenetic signals, with a high proportion of unresolved nodes (79.7% for promoter sequences and 51.4% for amino acid sequences), exceeding the recommended threshold of 30% for reliable phylogenetic resolution [33]. This high level of unresolved topology may be due to the diverse evolutionary backgrounds of the selected BR-signaling genes. Further analysis showed significant heterogeneity in the promoter sequences (Fig. 3B) and comparatively lower heterogeneity in the amino acid sequences (Fig. 4B). Despite these limitations, phylogenetic trees of the promoter and protein sequences were constructed to explore the evolutionary relationships between these genes.

Phylogenetic analysis of 1.5 kb upstream promoter sequences grouped the BR signaling genes into five distinct clades (Groups I-V). Group I was the largest, comprising 18 gene promoters, followed by Group IV, which comprised 11 gene promoters. Groups II and III were the smallest, containing only three promoters (Fig. 3C). In contrast, phylogenetic analysis of the amino acid sequences was divided into three groups (Group 1-III). Group I was the largest group, comprising 18 proteins, followed closely by Group II, with 17 proteins. Group III was the smallest group, comprising six proteins (Fig. 4C). Despite a high proportion of unresolved nodes, we observed considerable heterogeneity among the promoter sequences, suggesting divergent regulatory evolution (Fig. 3C). In contrast, the protein sequences showed the highest degree of similarity **(**Fig. 4 C). The similarity among non-homologous gene promoters and protein sequences may indicate convergent evolution or functional conservation driven by shared enzymatic or signaling roles in the BR signaling pathway.

### Analysis of conserved motifs and identification of cis-regulatory elements within the 1.5 kb promoter region of BR signaling genes

**Fig. 5:**
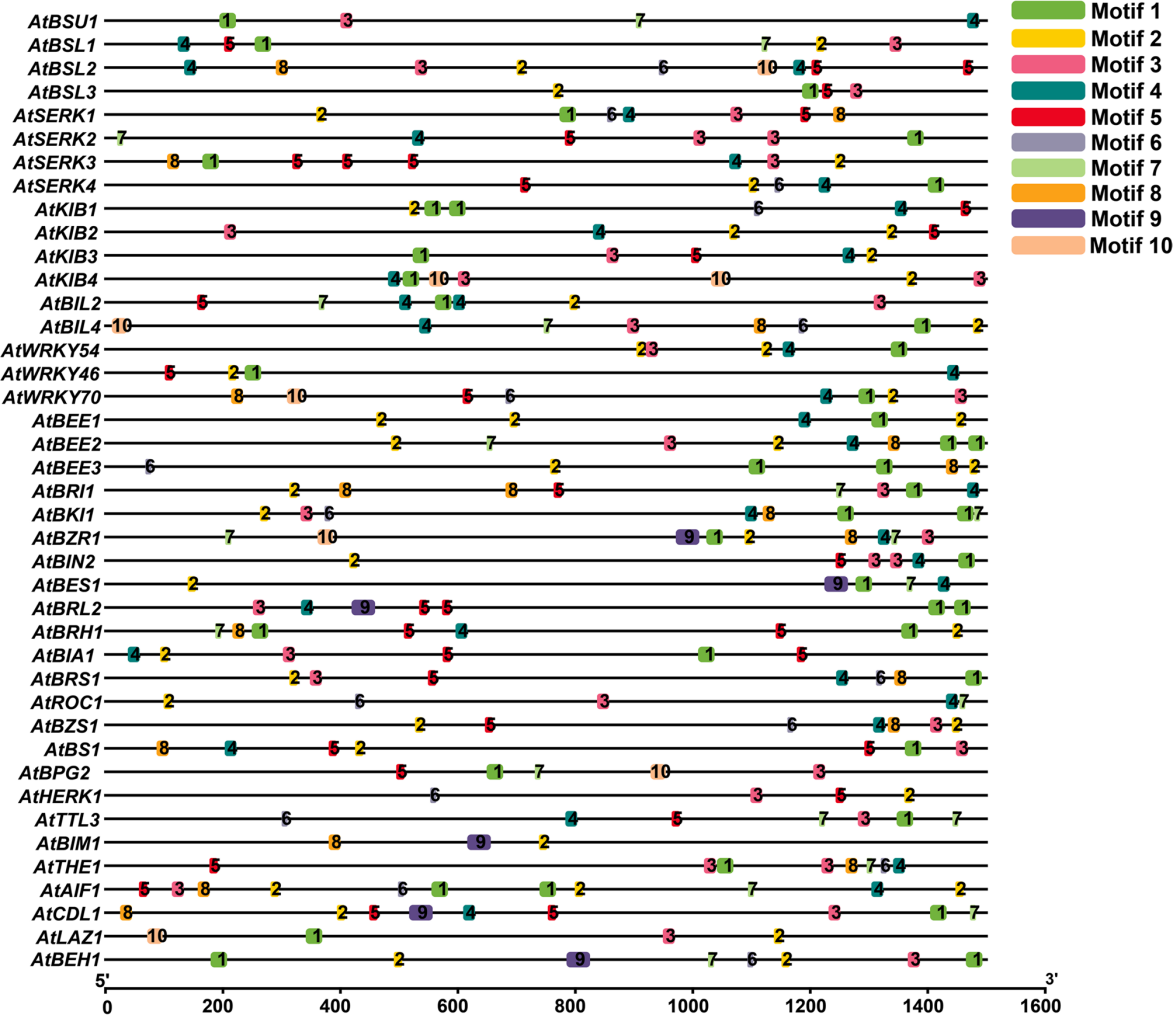
Identification of conserved motifs within 1.5 kb upstream promoter sequences of selected BR signaling genes in *Arabidopsis*

We analyzed the promoter sequences within the 1.5 kb upstream region of 41 BR signaling gene promoters for the presence of conserved sequence motifs. The analysis showed that motifs 2 and 3 were highly conserved in all 41 BR signaling gene promoters. Motifs 4, 5, and 7 were identified in 39 BR signaling gene promoters, with the exceptions of *AtHERK1*, *AtBIM1* (motif 4), *AtBSU1*,* AtHERK1* (motif 5), and *AtBZS1 and AtSERK4* (motif 7) (Fig. 5). Furthermore, motifs 1 and 2 were detected in 92.6% and 85.3% of promoters, respectively. In contrast, motifs 8, 9, and 10 were found to present in the least significant 43.9%, 12.1%, and 17.07%, respectively (Table S2). These findings suggest that variability in the distribution and frequency of conserved promoter motifs may contribute to differential gene regulation across plant developmental stages and in response to various biotic and abiotic stresses.

**Fig. 6:**
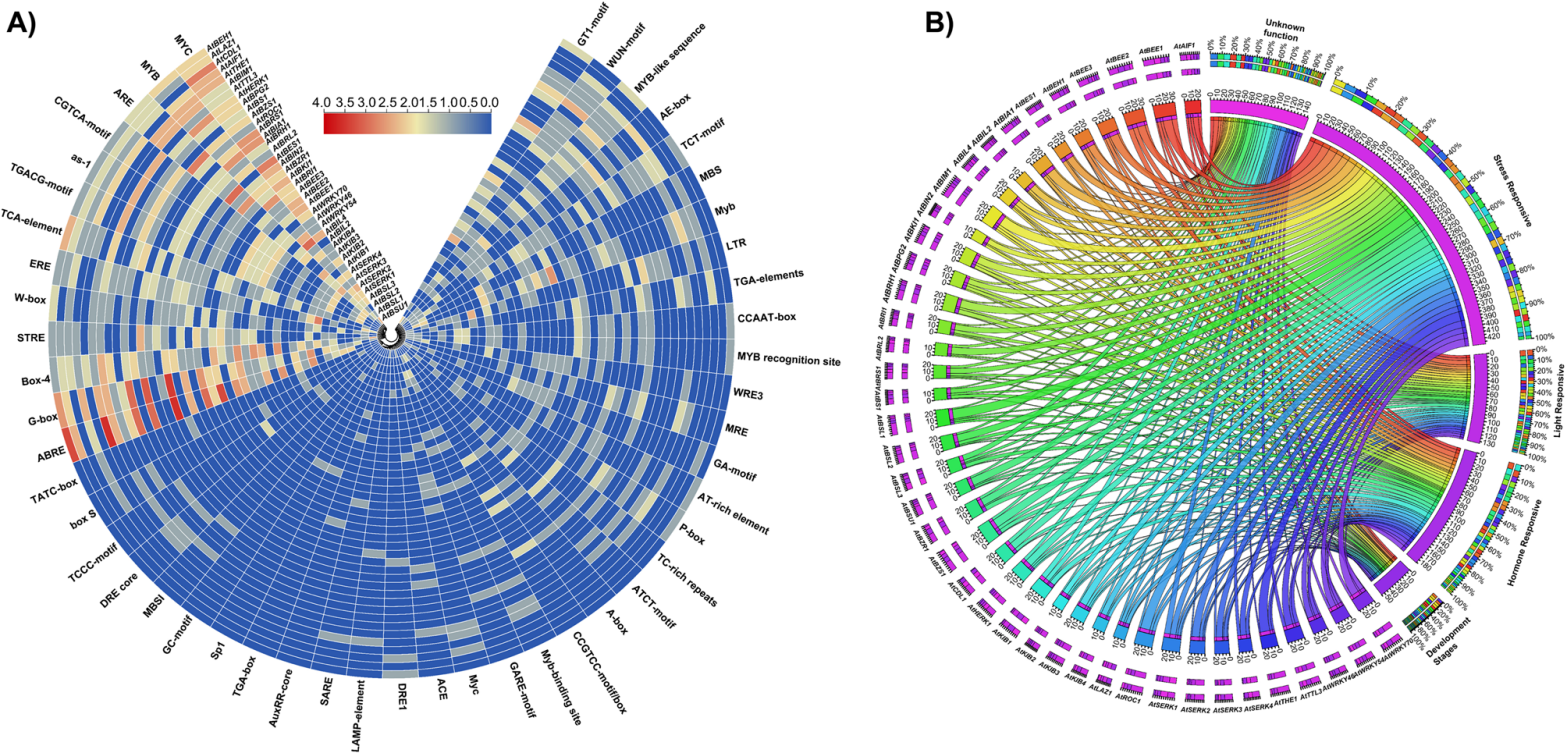
Identification of different cis-elements within 1.5 kb upstream promoter sequences of selected BR signaling genes in *Arabidopsis*. **A** The presence of different cis-elements in each gene promoter. **B** Occurrence of different cis-elements in each category within each gene promoter

Using the Plant CARE database, we analyzed the 1.5 kb upstream promoter regions of *Arabidopsis* BR signaling genes to identify cis-acting regulatory elements associated with plant development and hormone, light, and stress responses (Table S3). A total of 6057 cis-regulatory sites, representing 90 key cis-elements, were identified across 41 promoter regions. Among these, core cis-elements, such as TATA and CAAT boxes, were also present in the BR signaling genes (Table S4). In addition, 13 cis-elements (*MYC*,* MYB*,* ARE*,* CGTCA-motif*,* as-1*,* TGACG-motif*,* TCA-element*,* ERE*,* W-box*,* STRE*,* Box-4*,* G-Box*, and *ABRE*) were found to be significant in the promoter regions, highlighting their likely involvement in regulatory responses to environmental stimuli (Fig. 6A). Moreover, variations were observed in the total number of cis-regulatory elements in each BR signaling gene promoter. For example, the highest number of cis-elements was found in *AtBRH1 (205)* and the lowest in the *AtSERK1 (95)* gene promoters. These variations suggest that the differential expression of BR signaling genes during plant growth and in response to different environmental cues may be modulated by the diversity and distribution of cis-acting regulatory elements within the promoter regions.

The identified cis-acting elements were classified into five major categories: stress-responsive, hormone-responsive, light-responsive, development-associated, and those with unknown functions (Fig. 6B). Among these, stress-responsive cis-elements account for almost 32% of all identified cis-elements in *Arabidopsis*, which represent nearly one-third of the total cis-elements. The targeted promoter sequences revealed considerable variation in a wide range of stress-responsive elements, ranging from the highest in *AtBZS1* (29) to the lowest in *AtBSL3* (10). The second most abundant group had unknown functions, constituting almost 22.2% of the total cis-elements. Notably, cis-elements with unknown functions represent promising candidates for future functional characterization, particularly in the context of the BR-signaling pathway (Table S6). Hormone- and light-responsive cis-elements constituted the third most abundant category, accounting for 14.4% of the identified cis-elements. The frequency of hormone-responsive cis-elements ranged from the highest in *AtBRH1* (20) to the lowest in *AtKIB2 (02).* Similarly, light-responsive cis-elements were most frequent in *AtROC1* (14), whereas no such elements were detected in the promoter region of *AtCDL* and *AtSERK4* gene promoters. The four representative categories were cis-elements related to different developmental stages, with frequencies ranging from 0 to 5 across the gene promoters. These findings suggest that the functional roles of BR signaling genes might be influenced by their biological function by the presence and distribution of specific cis-elements in their regulatory regions.

### Identification of different TFbs within the 1.5 kb promoter region of BR signaling genes

**Fig. 7:**
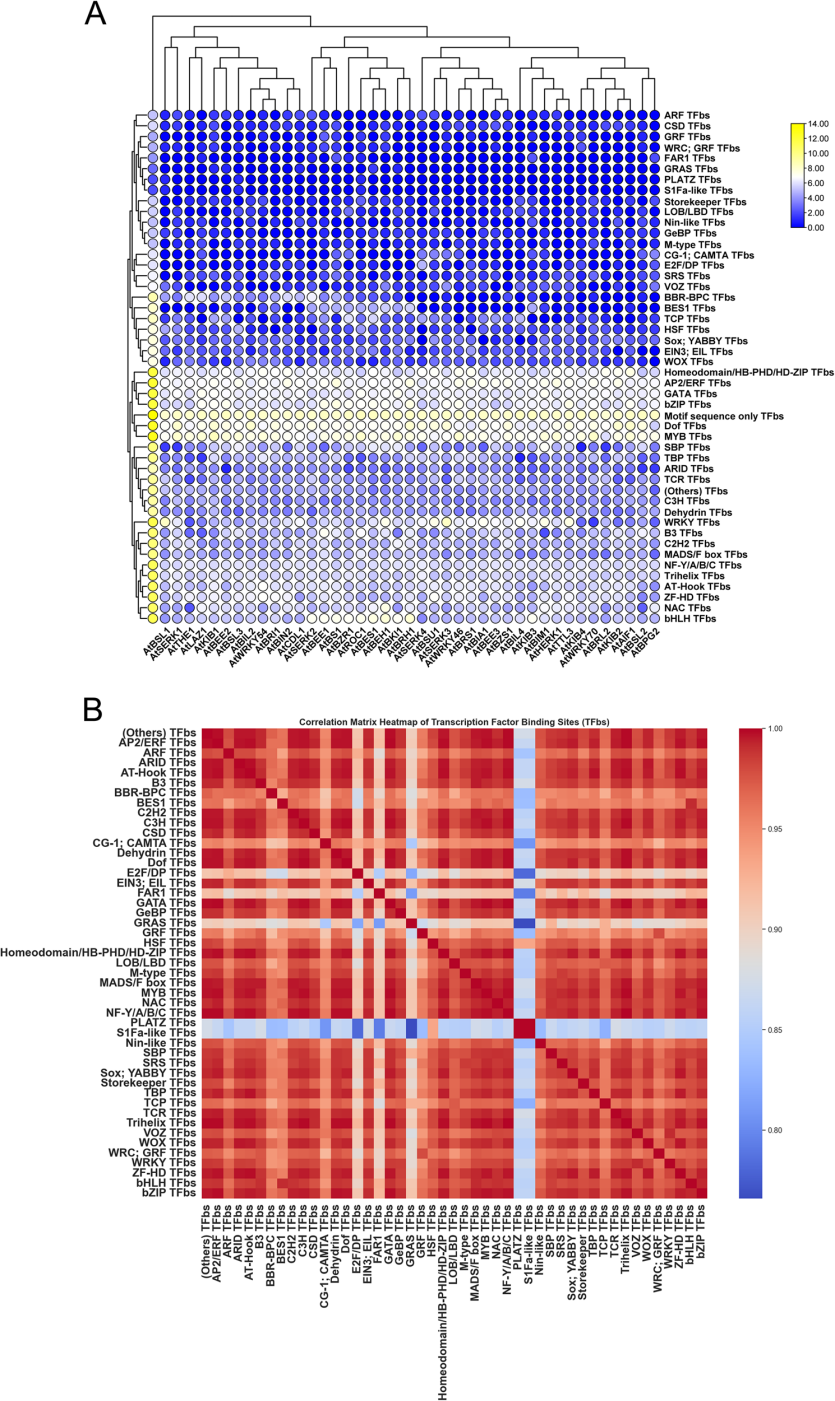
Identification of different TFbs within 1.5 kb upstream promoter sequences of selected BR signaling genes in *Arabidopsis*. **A** Occurrence of different TFBs within each gene promoter **B** Correlation matrix heatmap of different TFbs within 1.5 kb upstream promoter sequences

The 1.5 kb upstream promoter sequences of selected BR signaling genes were used to identify different transcription factor binding sites (TFbs) using PlantPAN 3.0. A total of 68,502 TFbs were detected, representing 48 distinct transcription families (Tables S5 and S6). These transcription families are known to regulate a wide range of biological processes, including plant growth, development, and responses to biotic and abiotic stresses, and may serve as key regulators for exploration in connection with BR signaling genes in *Arabidopsis*. The analysis revealed that MYB TFbs (7186), Dof TFbs (6256), AP2/ERF TFbs (4790), Homeodomain/HB-PHD/HD-ZIP TFbs (3541), bZIP TFbs (3724), GATA TFbs (3524) were found to be most abundant TFbs, which were widely distributed across 41 BR signaling genes in *Arabidopsis*. In addition, a substantial number of motif sequence-only TFbs (12089) were also detected, suggesting a potential target for further exploration in elucidating transcriptional regulation associated with BR signaling (Fig. 7A**)**. Interestingly, the overall distribution patterns of TFbs were consistent across all 41 BR-signaling genes. The frequency of TFbs ranged from highest in *AtBRH1* (2193) to lowest in *AtBPG2* (1244). Furthermore, several TFbs were present in very low frequency, such as GRAS TFbs (6), PLATZ TFbs (3), and S1Fa-like TFbs (3) (Fig. 7A). Although low in frequency, these TFbs may play specialized or context-dependent regulatory roles, making them promising candidates for future functional studies. A correlation matrix heatmap was generated to investigate the regulatory dynamics of the most common TFbs identified in the selected BR signaling genes in *Arabidopsis*. The analysis revealed that some TFbs were highly positively correlated, whereas some were less correlated with each other (Fig. 7B). In addition, TFbs that showed a high correlation may contribute to the regulation of BR signaling genes. Moreover, the observed variation in TFbs frequency in each gene promoter (highest and lowest) supports the hypothesis that the transcriptional regulation of BR signaling genes is, at least in part, influenced by the specific composition and abundance of TFbs. Further experimental validation is needed to determine the biological significance of BRs signaling genes with high and low TFb numbers.

### Gene expression analysis of selected BR signaling genes in different plant tissues and in response to different hormones and abiotic stresses

**Fig. 8:**
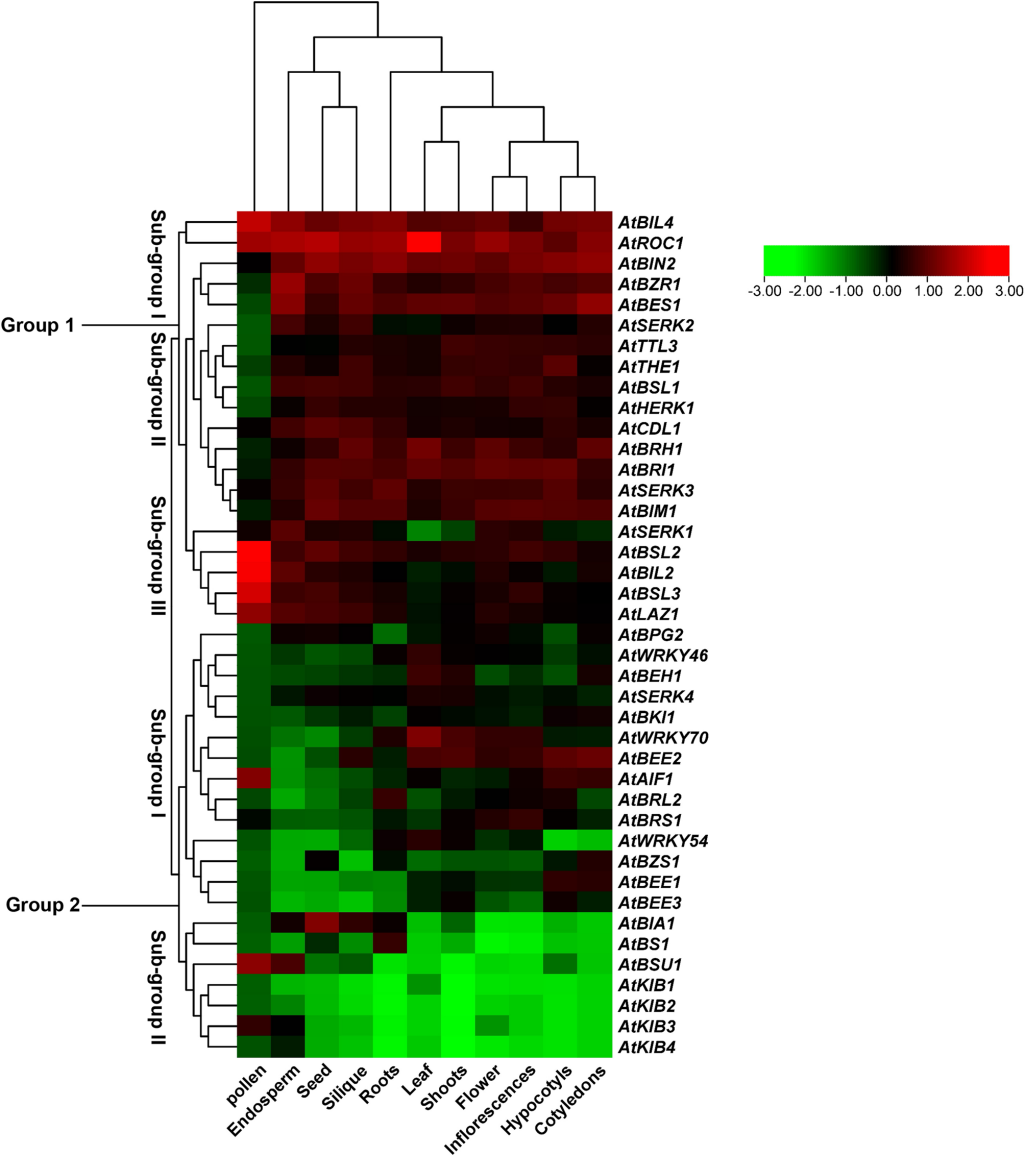
Expression patterns of 41 BR signaling genes in response to different plant organs

Based on the data retrieved (RNA-seq) from Public RNA-seq Databases, we analyzed the expression patterns of 41 BR signaling genes in *Arabidopsis* at different plant growth stages and their responses to various abiotic and hormonal stresses. Based on hierarchical clustering analysis, the expression data across different plant organs grouped the genes into two main groups (Groups 1 and 2) and further divided them into subgroups. Group 1 comprised 20 BR signaling genes and was divided into three subgroups (I, II, and III), whereas Group 2 included 21 BR signaling genes and was divided into two subgroups (I and II) (Fig. 8). Based on the results for Group 1, genes belonging to subgroup 1 (*AtBIL4*,* AtROC1*,* AtBZR1*,* AtBIN2*,* and AtBES1*) exhibited high expression across all plant tissues, except in pollen. In contrast, genes belonging to subgroup II of Group 2 (*AtBIA1*,* AtBS1*,* AtBSU1*,* AtKIB1*,* AtKIB2*,* AtKIB3*,* and AtKIB4*) were downregulated in all plant tissues, except in pollen (Fig. 8). Furthermore, genes belonging to other subgroups showed intermediate or tissue-specific expression patterns, suggesting that BR signaling gene activity is tightly regulated during plant development and may be organ specific.

**Fig. 9:**
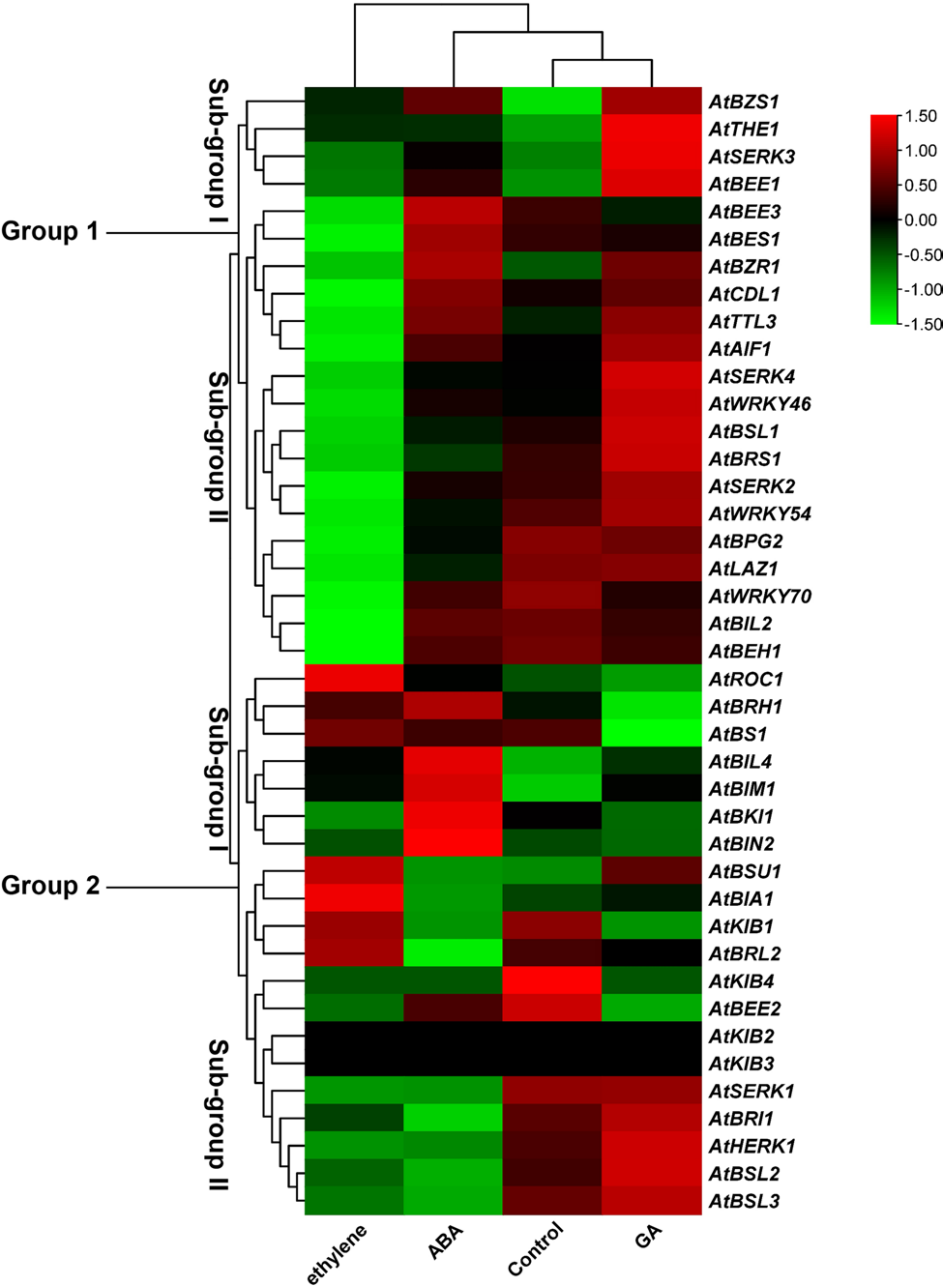
Expression patterns of 41 BR-signaling genes in response to different hormonal stresses

On the basis of hierarchical clustering analysis, the expression patterns of 41 BR signaling genes under various hormonal stress conditions were categorized into two major groups: Group 1 consisted of 21 and Group 2 comprised 20 BR signaling genes. Each group was further divided into subgroups (I and II) (Fig. 9). The results showed that genes within subgroups I and II of Group 1 exhibited high expression patterns in response to GA treatment, whereas genes in subgroup II of Group 1 were downregulated in response to ethylene. In contrast, genes in Subgroup I of Group 2 were upregulated under abscisic acid (ABA) treatment, whereas those in Subgroup II showed increased expression in response to GA. The remaining groups showed intermediate or no expression under hormonal stress and control conditions (Fig. 9).

**Fig. 10:**
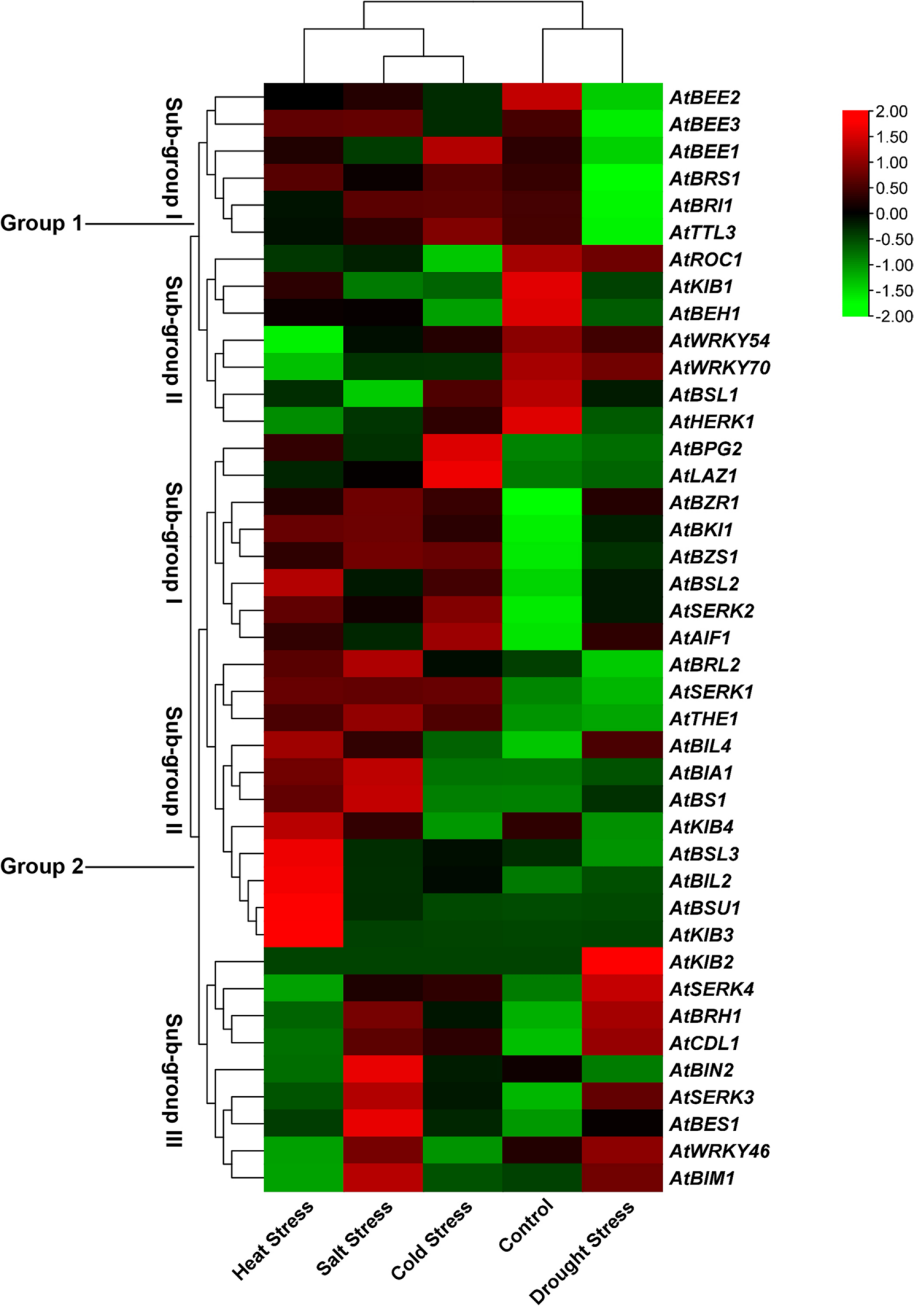
Expression patterns of 41 BR signaling genes in response to different abiotic stresses

Additionally, 41 BR signaling genes in response to different abiotic stresses were classified into two groups: Group 1 consisted of 13 genes, and Group 2 comprised 28 genes. These two groups were further subdivided into subgroups: Group 1 was split into two subgroups (I and II), and Group 2 was split into three subgroups (I, II, and III) (Fig. 10). The results showed that genes in both Subgroups I and II of Group 1 were consistently downregulated under drought stress. A similar downregulation pattern was observed under control conditions for genes belonging to subgroup I of Group 2. The analysis revealed that only a few genes were highly expressed in response to specific abiotic stresses; for instance, *AtBSL3*,* AtBIL2*,* AtBSU1*, and *AtKIB3* were upregulated under heat stress, *AtKIB2* under drought stress, and *AtBES1* and *AtSERK3* under salt stress. The remaining genes were not expressed under control or stress conditions (Fig. 10). These patterns suggest that a subset of BR signaling genes are specifically responsive to individual abiotic stress cues, reflecting their potential regulatory roles in stress adaptation mechanisms.

**Fig. 11:**
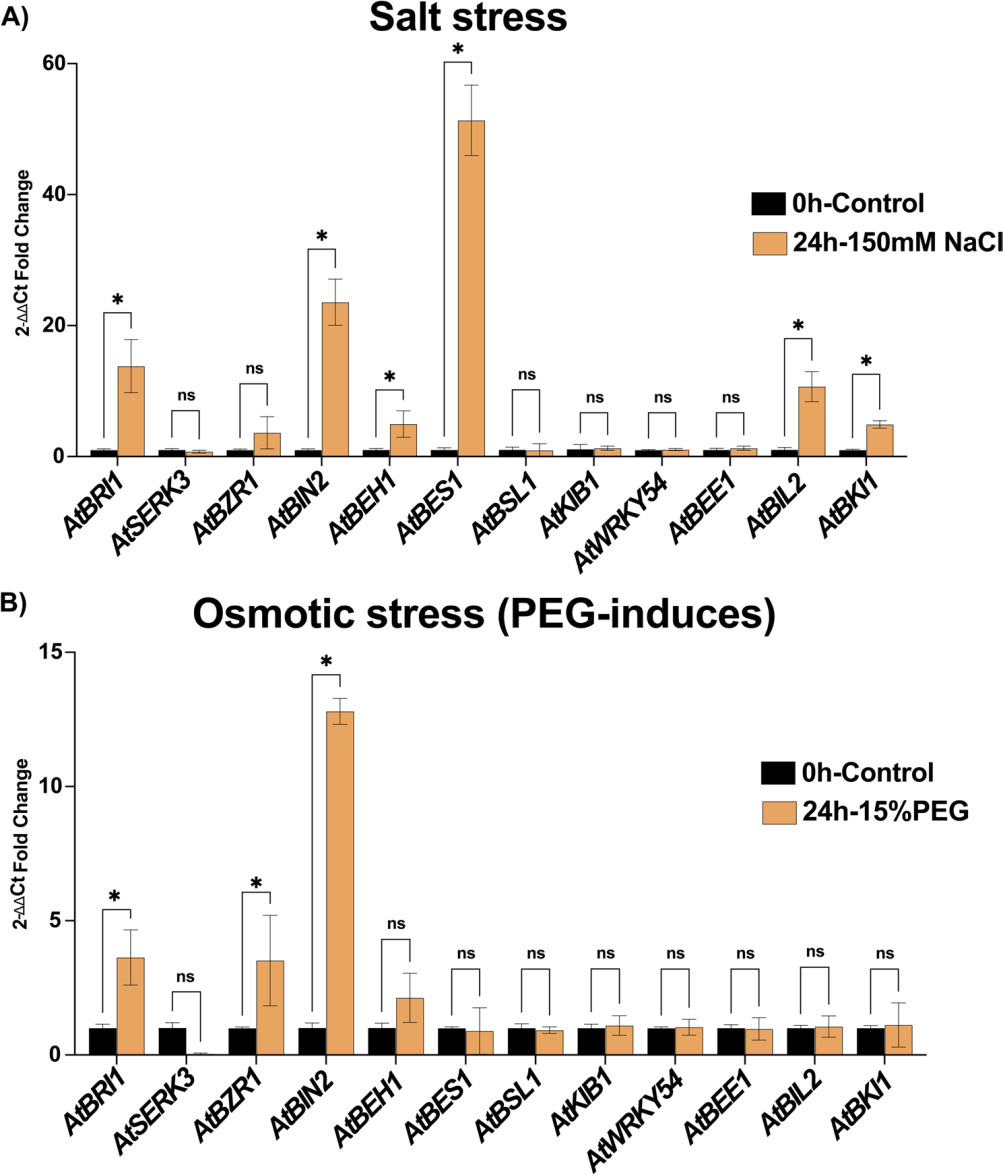
Validation ofexpression profiling using qPCR in response to **A**) Salt and **B**) Drought stress

Furthermore, to validate the expression patterns, qRT-PCR was performed on the main regulators of the BR signaling pathway and several BR signaling genes based on the expression patterns observed in RNA-seq data associated with bibliometric analysis and gene family member associations (*AtBRI1*,* AtSERK3*,* AtBZR1*,* AtBIN2*,* AtBEH*,* AtBES1*,* AtBSL1*,* AtKIB1*,* AtWRKY54*,* AtBEE1*,* AtBIL2*,* and AtBKI1*) under osmotic stress (PEG)-induced) and salt stress conditions compared to the control in *Arabidopsis* (ecotype Col-0). These 12 genes showed consistently high citation metrics and frequently appeared in BR signaling-focused literature. Moreover, the selected genes demonstrated strong and dynamic changes in expression, further supporting their functional importance. The qRT-PCR results revealed that *AtBRI1*, *AtBIN2*,* AtBEH1*,* AtBES1*,* AtBIL2* and *AtBKI1* genes were upregulated in response to salt stress conditions (Fig. 11A), whereas the remaining genes showed no significant change in expression under salt stress. These qRT-PCR results were generally consistent with the RNA-seq-based expression profiles, although some discrepancies were observed. For example, *AtBES1 and AtBIN2* consistently exhibited high expression during salt stress in both datasets, confirming its robust response. In contrast, *AtBRI1*,* AtBEH1*, *AtBIL2* and *AtBKI1* showed no changes in the RNA-seq data but were highly expressed under salt stress in qRT-PCR analysis (Figs. 10 and 11). In response to osmotic stress, *AtBRI1*,* AtBZR1and AtBIN2* were significantly upregulated, whereas remaining genes did not show any significant changes in expression patterns (Fig. 11B). Under osmotic stress, genes exhibited contrasting expression pattern between qRT-PCR and RNA-seq data. The differences between the qRT-PCR results and those shown in Fig. 10 may be attributed to variations in experimental conditions, tissue sampling, stress duration, or limitations of transcriptome datasets compared to targeted gene expression assays. Overall, results of our qRT-PCR confirmed the RNA-seq expression patterns and validated the importance of our selected genes supporting the robustness of our bibliometric and gene family-based selection strategy for key BR signaling regulators under osmotic and salt stress conditions.

### Analysis of protein-protein interaction of selected 41 BR signaling proteins

**Fig. 12:**
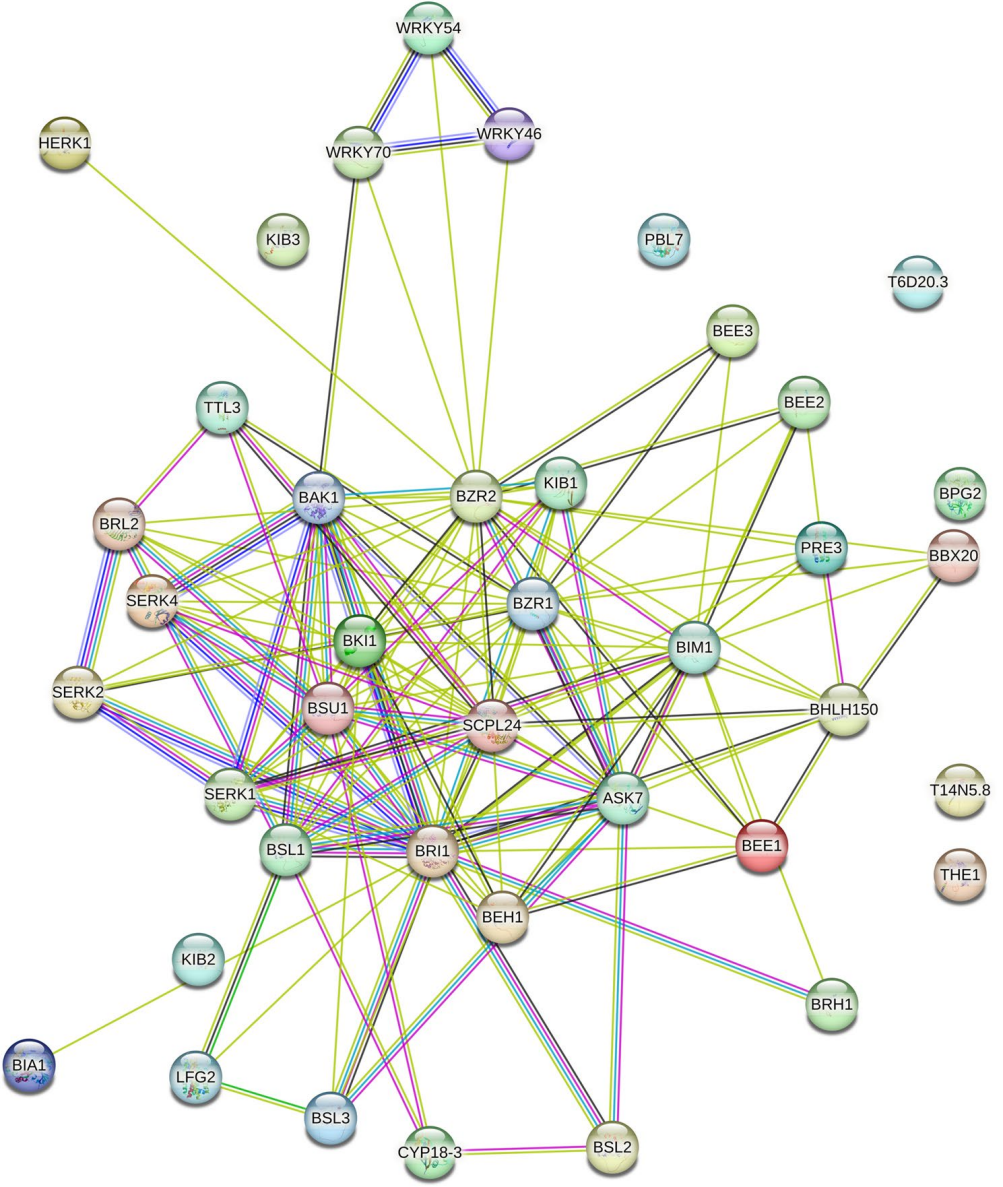
Protein-protein interactions (PPI) of selected BR signaling proteins in *Arabidopsis*

Protein-protein interactions (PPI) among the enzymes encoded by the 41 BR signaling genes were predicted using the STRING 11.5 database to explore the regulatory complexity of BR signaling. The results revealed that 34 out of 41 encoded proteins showed strong interaction networks with an enrichment *p*-value < 0.01, except for AtKIB3, AtKIB2, AtBPG2, AtTHE1, AtLAZ1, AtBIL2, and AtCDL1 (Fig. 12). The network exhibited an average node degree of 6.98 and an average local clustering coefficient of 0.562, indicating dense interactions. Most proteins formed extensive interaction hubs, except for AtBIA1 and AtHERK1, which interacted only with AtBRI1 and AtBZR2, respectively. A detailed annotation of all predicted interactions for each BR signaling protein is provided in Table S7. These findings demonstrate the complexity of the BR signaling network and highlight the versatility of proteins in coordinating plant growth, developmental processes, hormone signaling, and responses to environmental stresses.

## Discussion

Several studies have been conducted on in-silico approaches to analyze, characterize, and evaluate the expression of various gene families involved in regulating plant developmental stages and responses to environmental stresses, particularly in the model dicot and monocot plants [34–39]. These studies have revealed the presence and functional significance of different cis-regulatory elements and TFbs, which are critical for gene regulation in response to specific targets of interest. For instance, a comprehensive in-silico analysis of 39 BR signaling genes in rice revealed intricate regulatory mechanisms and gene interdependencies associated with growth, development, and abiotic stress tolerance [28]. Although significant progress has been made in understanding the BR signaling pathway, the complexity of this pathway in *Arabidopsis*, especially due to the central roles of key transcription factors such as *BES1* and *BZR1*, which regulate the expression of thousands of largely uncharacterized genes, has restricted a systematic genome-wide analysis in this species. To address this gap, we conducted a comparative and detailed in-silico analysis of 41 BR signaling genes in *Arabidopsis* using various bioinformatics tools and validated the expression of key regulators and selected BR signaling genes belonging to the same gene families using the qRT-PCR protocol. Our findings offer novel insights into the regulatory architecture of BR signaling in *Arabidopsis*, providing a valuable framework for interpreting BR-mediated transcriptional networks in other dicot species.

In our phylogenetic analysis, the promoters showed a high unresolved ratio of 79.7%, indicating low sequence conservation among the promoters. In contrast, the amino acid sequence-based phylogeny exhibited a lower unresolved ratio of 51.4% and demonstrated greater sequence similarity, suggesting a higher level of evolutionary conservation at the protein level. Similar low promoter sequence conservation patterns have previously been observed in rice BR signaling genes [28], which may reflect the diverse gene family origins of the selected genes. These phylogenetic relationships provide insights into the evolutionary divergence and functional specialization of BR signaling genes, which can be classified into distinct gene families based on sequence homology. Moreover, a significant variation in the distribution of conserved motifs was observed among the 41 BR signaling gene promoters. Motifs 1 and 2 were present in all gene promoters, indicating that variation in the distribution of conserved motif genes may influence gene expression patterns [40].

This study identified diverse distribution patterns of cis-acting regulatory elements representing key cis-elements within the 1.5 kb upstream promoter region of 41 BR-signaling gene promoters in *Arabidopsis*. In rice, key cis-regulatory elements have also been identified in 39 BR signaling gene promoters, including stress-responsive elements, followed by hormones and light-responsive cis-elements, most of which are represented in their promoter regions [28]. *AtBRH1*, the most represented cis-element, encodes a novel RING finger protein that alters leaf morphology with thicker stem inflorescences, suggesting that it might play a role in later hormonal effects [41, 42]. In line with its regulatory complexity, *AtBRH1* also exhibits many hormone-responsive cis-elements, suggesting a role in hormone-mediated signaling pathways. Consistent with this, gene expression analysis in the present study indicated that *AtBRH1* was highly upregulated in response to drought stress and ABA treatment (Figs. 9 and 10). This strong expression response may be attributed to the presence of more significant cis-acting elements. In particular, *AtBRH1* has emerged as a promising candidate for future functional studies aimed at elucidating its role in the crosstalk between ABA and BR signaling and its potential involvement in drought stress tolerance mechanisms [42].

In contrast, *AtSERK1* is not influenced by any cis-acting element in response to any plant organ or stress. This may be due to its established role in the early stages of somatic embryogenesis rather than in abiotic stress responses [43]. Moreover, the highest number of stress-responsive elements was observed in *AtBZS1*, supporting previous findings that associate this gene with abiotic stress tolerance mechanisms [44]. Similarly, light-responsive elements were highly present in the *AtROC1* gene promoter, which is highly expressed in leaf tissues and under ethylene hormonal treatment and is downregulated in response to cold stress. Interestingly, *AtROC1* has been reported to confer cold tolerance in *Arabidopsis* by modulating jasmonic acid signaling [45], confirming its complex role in light and hormonal signaling integration. The second most abundant cis-elements were found to have unknown functions across all promoters and could be potential targets for functional characterization, particularly in the context of BR signaling genes. These findings suggest that the biological roles and expression patterns of BR signaling genes are likely influenced by the type and frequency of cis-regulatory elements present in their promoters.

We also analyzed TFbs in selected 41 BR signaling genes, and it is important to note that TF families are classified mostly according to their respective pathways. The identified TFbs are involved in various functions during plant growth and play key roles in the response to environmental stresses. In rice, TCP, WRKY, bHLH, NAC, BES1, bZIP, MYB, GATA, and AP2/ERF TFbs were identified as the most abundant TFbs in the promoter regions of BR signaling genes [28]. However, in the present study, MYB, Dof, AP2/ERF, Homeodomain/HB-PHD/HD-ZIP, bZIP TFbs, and GATA TFbs were found to be the most significant TFbs for BR signaling genes in *Arabidopsis*. Interestingly, MYB TFbs were the most abundant in *Arabidopsis* and ranged from the highest *AtBRH1* (also containing the most cis-regulatory elements) to the lowest in *AtBPG2* genes. However, TCP TFbs were significantly higher in rice than in all TFbs, particularly in the *OsMADS22* and *OsSMOS1* gene promoters [28]. MYB TFs are well-established regulators of nearly all aspects of plant growth and development, including responses to abiotic and biotic stresses. They are crucial for activating plant defense mechanisms, contributing to stress tolerance and overall plant fitness [46, 47].

In contrast, several TFbs, such as GRAS TFbs, PLATZ TFbs, and S1Fa-like TFbs, were detected at a very low frequency, which made it possible to investigate their role in response to different aspects of plant biology. We concluded that the expression of BR signaling genes is closely linked to the presence and frequency of specific TFbs in the promoters. However, further experiments are needed to determine the biological significance of BRs signaling genes with high and low TFb numbers, particularly under various developmental and stress conditions.

The expression patterns of the 41 selected BR signaling genes showed significant variation in *Arabidopsis.* Based on hierarchical clustering analysis, most genes showing similar expression patterns and being closely related to each other were grouped, indicating that they may have similar biological functions during plant growth, development, and responses to different stresses [48]. For example, in Group 1, genes belonging to subgroup 1, such as *AtBIL4*,* AtROC1*,* AtBZR1*,* AtBIN2*,* and AtBES1*, were highly expressed across all plant tissues, except in pollen. In contrast, genes classified within subgroup II (*AtBIA1*,* AtBS1*,* AtBSU1*,* AtKIB1*,* AtKIB2*,* AtKIB3*,* and AtKIB4*) were consistently downregulated in all plant tissues, except for pollen. However, *AtBIN2* is known to interact with key transcription factors, such as *AtBES1* and *AtBZR1*, which regulate the expression of other BR-responsive genes and lead to the activation or repression of downstream targets, thereby fine-tuning the BR signaling cascade [49]. This tissue-specific expression pattern may reflect specialized roles in reproductive development or indicate functional redundancy outside pollen-specific processes. In another study, *AtBZR1 and AtBES1* were highly expressed during seed formation and improved seed size and quality [50]. Furthermore, the expression of BR signaling genes is regulated by the presence, type, and frequency of cis-regulatory elements within their promoter regions, which influence their response to developmental stages and environmental cues [51].

Moreover, genes belonging to subgroups I and II of Group 1 showed high expression patterns in response to GA, whereas genes belonging to subgroup II were downregulated under ethylene treatment. Similarly, genes belonging to subgroup I of Group 2 were upregulated in response to ABA, whereas those in subgroup II showed increased expression following GA exposure. These findings support previous reports suggesting that GA and BR pathways interact at the signaling level [52]. In this model, DELLA proteins are known to physically associate with positive regulators of BR signaling, such as BZR1, thereby increasing BR signaling gene expression in response to GA [52–54]. Moreover, the crosstalk between BR and ethylene signaling pathways remains unclear; however, the results of the present study indicate a potential hint for future research to investigate the connection between BR and ethylene-responsive genes [55]. Under abiotic stress, genes belonging to subgroups I and II of Group 1 were generally downregulated. However, several BR signaling genes were highly expressed under specific stress conditions, such as *AtBSL3*,* AtBIL2*,* AtBSU1*, and *AtKIB3* under heat stress, *AtKIB2* under drought stress, and *AtBES1* and *AtSERK3* under salt stress. Recently, the expression of genes of the BES1 gene family was analyzed under drought conditions in two chickpea cultivars, and it was reported that most BR signaling genes were upregulated under drought conditions [56]. The high number of stress-responsive cis-regulatory elements and TFbs in the promoters of these genes likely contribute to their dynamic expression patterns under abiotic stress conditions.

qRT-PCR results did not fully align with the RNA sequencing database analysis, which may be attributed to differences in experimental conditions, including developmental stages, tissue specificity, and stress exposure protocols. Moreover, BR signaling is tightly regulated at multiple levels, including post-transcriptional and post-translational mechanisms, which may not be detected equally by both methods. For example, specific BR-responsive genes may undergo alternative splicing or post-translational modifications under stress conditions, which can affect their detection and quantification [57]. Moreover, certain BR components display dual regulatory roles, such as *BIN2*, which can activate one protein while simultaneously destabilizing another, thus offering a mechanism for integrating BR signaling with the transcriptional network [58]. Several studies have reported the role of BR-related genes (both positive and negative) in response to salt and drought stress [59]. For example, overexpression of the BR biosynthetic gene *AtDWF4* enhanced drought stress resistance in *Brassica napus* [60]. Similarly, overexpression of *SoCYP85A1 in Spinacia oleracea* improved drought tolerance [61]. Moreover, the BR signaling gene *BdBRI1* in *Brachypodium distachyon* and *SlBRI1* mutants in tomatoes have been associated with drought tolerance [62, 63].

In contrast, mutants of *bes1-D* with increased BR signaling showed decreased drought tolerance, whereas bri*1-301* mutants showed increased drought tolerance and decreased BR signaling in *Arabidopsis* [64, 65]. Moreover, our findings related to salt stress were further supported by studies on the overexpression of the BR signaling gene *SiBZR1D*, which enhances BR signaling and increases salt tolerance in both tomato and *Arabidopsis* [66]. Conversely, in another study, the *det2-1* and *bin2-1* mutants were more sensitive to salt stress than the control (WT) in *Arabidopsis*, indicating a positive role for endogenous BRs in salinity tolerance [67]. These findings collectively highlight the significance of BR signaling in abiotic stress responses. Thus, integrating various methods, such as qRT-PCR and RNA-seq, is crucial for validating expression patterns and achieving a comprehensive understanding of gene regulation in response to abiotic stress. Additional studies using targeted mutants and transgenic lines are needed to reveal the specific roles of individual BR-related genes in salt stress tolerance in *Arabidopsis* and across economically important crop species.

Moreover, PPI showed strong interactions, suggesting a high degree of functional connectivity among BR signaling components. A comparative analysis in rice revealed that,26 out of 39 BR signaling-related proteins exhibited strong interactions with each other [28]. A model of protein interactions involved in BR signaling pathways has been described for *Arabidopsis* and rice to understand the essential roles of these proteins in plant growth, development, and responses to environmental stresses [68, 69]. However, our analysis also provides novel predicted interactions that have not been previously reported. For example, AtBIA1 and AtHERK1 were predicted to interact only with AtBRI1 and AtBZR2, respectively. Such limited connectivity suggests potential specialized or context-dependent roles for these proteins. To validate and expand upon these predictions, detailed structural analyses of the corresponding protein complexes are needed, particularly under conditions with and without active BRs, to understand the dynamic nature of these interactions. Furthermore, PPI analysis revealed a subset of BR signaling proteins (AtKIB3, AtKIB2, AtBPG2, AtTHE1, AtLAZ1, AtBIL2, and AtCDL1) that did not interact with any other known BR components. These findings suggest the existence of uncharacterized regulatory modules or transient interactions that may require alternative experimental approaches, such as co-immunoprecipitation or yeast two-hybrid assays, to be fully explored. Together, this network-level view provides a foundation for future studies to dissect the functional architecture of BR signaling and its integration with other cellular pathways in plants.

## Conclusion

A detailed and comparative analysis of BR signaling genes revealed key insights into the structural, regulatory, and functional characteristics of *Arabidopsis*. Phylogenetic analysis showed high conservation at the protein level, but significant divergence was observed among promoter sequences, suggesting evolutionary adaptation in gene regulation. The identification of cis-elements and TFbs within the promoter regions of each BR signaling gene in *Arabidopsis has* revealed the complexity of BR-mediated transcriptional control. Furthermore, the identification of several novel and potential cis-elements (those with unknown functions) and TFbs (present at low frequencies, such as GRAS, PLATZ, and S1Fa-like) requires further investigation to reveal their putative roles in plant growth and development. The expression patterns of BR signaling genes, such as *AtBRI1*, *AtBIN2*, *AtBZR1*, and *AtBES1*,* are* differentially expressed across various plant developmental stages, hormonal treatments, and abiotic stresses. Moreover, the expression patterns of BR signaling genes, such as *AtBRH1* and *AtBZS1*, were influenced by the presence of more cis-elements and TFbs in their promoter regions. However, the precise molecular mechanisms associated with the regulatory expression of cis-elements and TFbs remain unclear and require further investigation. Overall, the findings of this study enhance our understanding of the dynamic regulation of BR-signaling genes and their functional versatility in coordinating growth, development, and environmental adaptation. 

## Supplementary Information

Supplementary Material 1: Figure S1 Gene structure of the selected BR signaling candidates.

Supplementary Material 2: Supplementary File S1: List of primers used in the qPCR method for expression validation.

Supplementary Material 3: Table S1: Gene annotations of 41 BR signaling genes in *Arabidopsis.*

Supplementary Material 4: Table S2: Detailed annotations of identified conserved motifs within 1.5 kb upstream promoter sequences of selected BR signaling genes in *Arabidopsis*.

Supplementary Material 5: Table S3: All identified cis-elements within 1.5 kb upstream of the promoter sequences of selected BR signaling genes in *Arabidopsis*.

Supplementary Material 6: Table S4: List of cis-elements in different categories.

Supplementary Material 7: Table S5: All identified TFbs within 1.5 kb upstream of the promoter sequences of selected BR signaling genes in *Arabidopsis*.

Supplementary Material 8: Table S6 List of different TFbs present in each gene promoter.

Supplementary Material 9: Table S7: Detailed information on PPI annotation of the selected BR signaling proteins.

Supplementary Material 10: Table S8: Detailed information about the RNA-seq data used in this study, including the corresponding project IDs from public database.

## Data Availability

The datasets used in this study are fully presented within the published article and its supplementary materials. The project IDs for the RNA sequence data used in the study are provided in Table S8.
