# Supplementary material for: Elucidating the role of brassinosteroid signaling genes and their promoters in Arabidopsis revealed regulatory mechanisms in plant development and responses to different abiotic stresses
Source: BMC Plant Biol. 2025 Jul 28;25:970. doi: 10.1186/s12870-025-06960-6 (PMC12302695; doi:10.1186/s12870-025-06960-6)
Supplement: Supplementary file 2 — Supplementary Material 2: Supplementary File S1: List of primers used in the qPCR method for expression validation. [file 12870_2025_6960_MOESM2_ESM.docx]

A.thaliana TAIR10|AT4G39400.1 CDS

Forward primer GCGAATTCAATCTCCGGTGC

Reverse primer CGGGAGAAATCGCCGGATAA

A.thaliana TAIR10|AT4G33430.2 CDS

Forward primer AGCGTATTGCGTTGGGATCT

Reverse primer GTGGAAAGGTACTCAGGGGC

A.thaliana TAIR10|AT4G39400.1 CDS

Forward primer GCGAATTCAATCTCCGGTGC

Reverse primer CGGGAGAAATCGCCGGATAA

A.thaliana TAIR10|AT3G50750.1 CDS

Forward primer GCCGTGTTCGTCAATCCAAC

Reverse primer AGCGAGGTTTCCAGACGAAG

1. thaliana TAIR10|AT4G18710.1 CDS

Forward primer TAGTTACATGGCGGAGCGAG

Reverse primer CGGATGATCCATCACACGCA

A.thaliana TAIR10| AT1G19350.1 CDS

Forward primer CCTGTCACTCCACCAGTGTC

Reverse primer AGACACCGCATAAAACGGGT

A.thaliana TAIR10| AT4G03080.1 CDS

Forward primer GTTCAAGGGGATGGTCCAGG

Reverse primer ACATGCCATCAGAACGAGCA

A.thaliana TAIR10| AT4G12810.1 CDS

Forward primer AAGTCTCTAGCAGGCGAAGC

Reverse primer ATGGTAGACGAAGACACCGC

A.thaliana TAIR10| AT2G40750.1 CDS

Forward primer GGTGGAGATTCCGGTGAGAG

Reverse primer TGCTTTGCATCCTTGCGTTG

A.thaliana TAIR10| AT1G18400.1 CDS

Forward primer ATCAGAGACTAGCGGCGTCT

Reverse primer GCTGTGGCTATCAGTGGCTT

A.thaliana TAIR10| AT2G42080.1 CDS

Forward primer TTAGAACGCAAACGTCGCAC

Reverse primer AGGTTTGCTTCCGGGATGAG

A.thaliana TAIR10| AT5G42750.1 CDS

Forward primer GACCTAAGTCACGCCGTCAA

Reverse primer CCGGAGCCGAGAACAATTCA
