# Supplementary figures and images for: Elucidating the role of brassinosteroid signaling genes and their promoters in Arabidopsis revealed regulatory mechanisms in plant development and responses to different abiotic stresses
Source: BMC Plant Biol. 2025 Jul 28;25:970. doi: 10.1186/s12870-025-06960-6 (PMC12302695; doi:10.1186/s12870-025-06960-6)

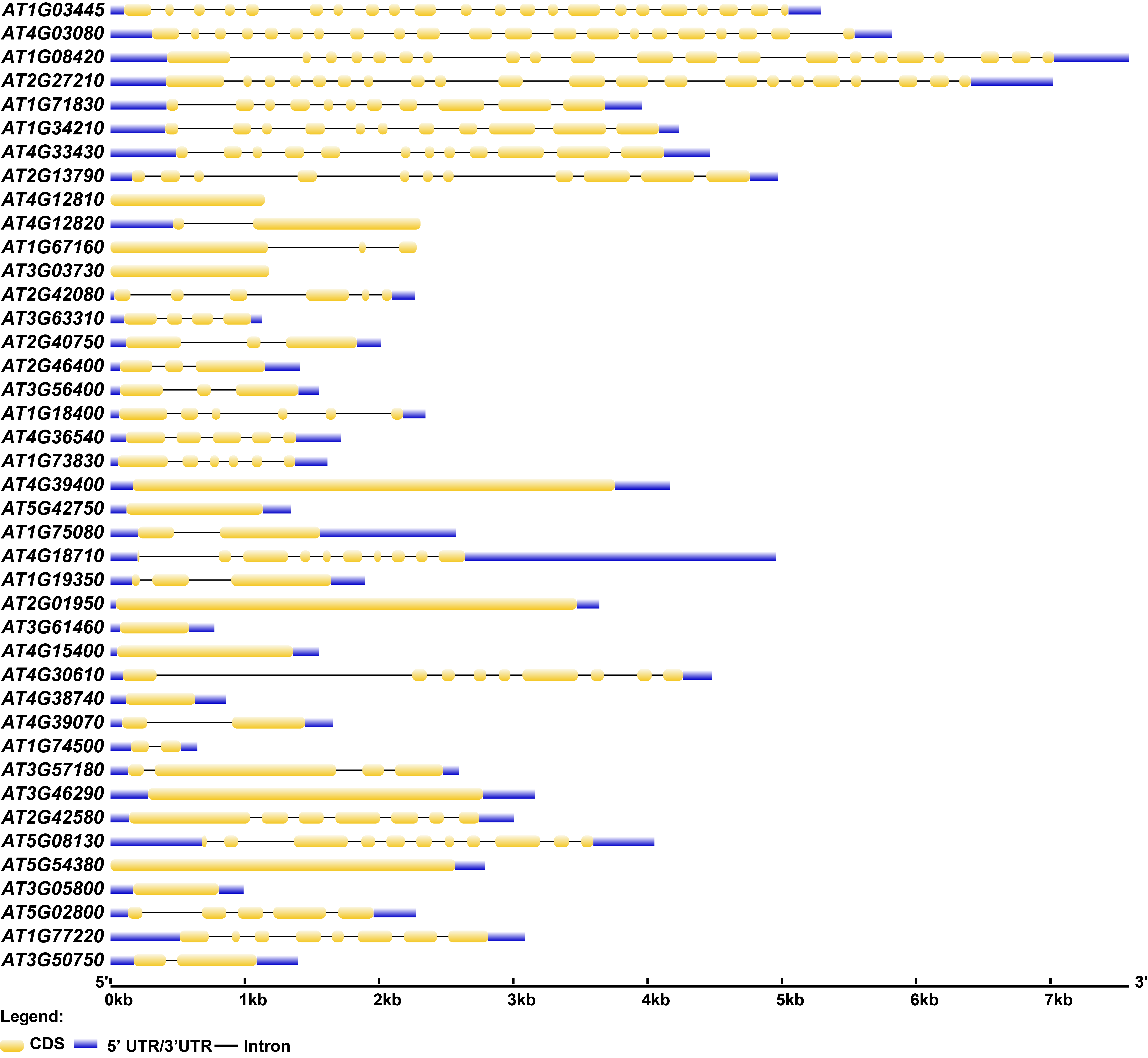

Supplement: Supplementary file 10 — Supplementary Material 10: Table S8: Detailed information about the RNA-seq data used in this study, including the corresponding project IDs from public database. [file 12870_2025_6960_MOESM10_ESM.jpg]
